# Circulating extracellular DNA is an independent predictor of mortality in elderly patients with venous thromboembolism

**DOI:** 10.1371/journal.pone.0191150

**Published:** 2018-02-23

**Authors:** Miguel Jiménez-Alcázar, Andreas Limacher, Rachita Panda, Marie Méan, Josephine Bitterling, Sven Peine, Thomas Renné, Jürg H. Beer, Drahomir Aujesky, Bernhard Lämmle, Tobias A. Fuchs

**Affiliations:** 1 Institute of Clinical Chemistry and Laboratory Medicine, University Medical Center Hamburg-Eppendorf, Hamburg, Germany; 2 CTU Bern, and Institute of Social and Preventive Medicine, University of Bern, Bern, Switzerland; 3 Division of General Internal Medicine, University Hospital of Bern and University of Bern, Bern, Switzerland; 4 Service of Internal Medicine, Lausanne University Hospital, Lausanne, Switzerland; 5 Institute of Transfusion Medicine, University Hospital Hamburg-Eppendorf, Hamburg, Germany; 6 Department of Molecular Medicine and Surgery, Karolinska Institute, Stockholm, Sweden; 7 Cantonal Hospital of Baden, Baden, and Molecular Cardiology, University Hospital of Zürich, Zürich, Switzerland; 8 Department of Hematology, University Hospital Bern and University of Bern, Bern, Switzerland; 9 Center for Thrombosis and Hemostasis, University Medical Center Mainz, Mainz, Germany; Institut d'Investigacions Biomediques de Barcelona, SPAIN

## Abstract

**Background:**

Venous thromboembolism (VTE) is a major cause of morbidity and mortality in elderly patients. Extracellular DNA is a pro-inflammatory and pro-thrombotic mediator *in vitro* and in animal models. Levels of circulating extracellular DNA (ceDNA) are increased in VTE patients, but the association of ceDNA with VTE extent and clinical outcome is poorly understood.

**Objectives:**

We analyzed the association of ceDNA with the extent of VTE, categorized as distal and proximal deep vein thrombosis and pulmonary embolism, and with the clinical outcomes VTE recurrence and mortality.

**Methods:**

We quantified ceDNA by a fluorescent probe, as well as circulating nucleosomes and neutrophil extracellular traps (NETs) by ELISA in plasma from 611 patients aged ≥ 65 years with acute VTE of a prospective cohort study (SWITCO65+).

**Results:**

Levels of ceDNA and nucleosomes, but not NETs, correlated with VTE extent. Infectious comorbidities independently increased ceDNA levels in VTE. CeDNA strongly correlated with C-reactive protein and leukocytosis, suggesting an association of ceDNA with inflammation in VTE patients. CeDNA furthermore predicted PE-related and all-cause mortality, but not VTE recurrence, during a 3-year follow-up.

**Conclusions:**

Our study suggests that ceDNA levels in VTE patients reflect the degree of inflammation and may serve as a biomarker to stratify VTE patients at risk for mortality.

## Introduction

Venous thromboembolism (VTE), the combined disease entity of deep vein thrombosis (DVT) and pulmonary embolism (PE), is a major cause of morbidity and mortality [1]. The incidence of VTE increases with age [2] and the majority of VTE patients are 65 years or older [3]. The increasing age of the population in Western countries necessitates new strategies for the stratification of patients at high risk for adverse outcomes of VTE.

Plasma contains circulating extracellular DNA (ceDNA) and levels of ceDNA increase in inflammatory, cardiovascular, and thromboembolic diseases [4, 5]. *In vitro* and *in vivo* data suggest an active role of ceDNA in inflammation and thrombosis. The complex of extracellular DNA with histones and neutrophil-derived peptides stimulates inflammation via activation of pattern recognition receptors on immune cells [6, 7]. In addition, extracellular DNA and histones contribute to blood clotting via activating coagulation factors and platelets [8, 9]. In agreement with these findings, infusions of DNases reduce tissue inflammation and prevent thrombosis in murine models by degrading extracellular DNA [10, 11]. Potential sources of ceDNA are dead cells and neutrophils through the release of neutrophil extracellular traps (NETs), which are characterized as DNA-fibers containing histones and enzymes from neutrophil granules [12].

CeDNA levels are increased in VTE patients, when compared to healthy controls [4, 13, 14]. However, the association of ceDNA with the extent and outcome of VTE is poorly understood. We quantified ceDNA as well as circulating nucleosomes and NETs in plasma from patients with acute VTE. Our data suggests that ceDNA in VTE patients reflects the magnitude of inflammation and may serve as a biomarker to stratify VTE patients at risk for mortality.

## Materials and methods

### The Swiss venous thromboembolism cohort 65+

The Swiss Venous Thromboembolism Cohort 65+ (SWITCO65+) is a prospective multicenter cohort study enrolling patients aged ≥ 65 years with acute, symptomatic VTE at nine Swiss university and non-university hospitals from 2009 to 2013 [15, 16]. Patients with acute VTE were identified in the inpatient and outpatient services of the study sites. Symptomatic DVT was diagnosed as the acute onset of leg pain or swelling plus incomplete compressibility of a venous segment on ultrasonography or an intraluminal filling defect on contrast venography [17]. Because iliac veins and the inferior vena cava are technically difficult to compress, additional diagnostic criteria for the iliac and caval DVT included abnormal duplex flow patterns compatible with thrombosis, an intraluminal filling defect on spiral computed tomography, or magnetic resonance imaging venography [18, 19]. Patients with isolated distal DVT were only eligible if the incompressible distal vein transverse diameter was at least 5 mm, because compression ultrasonography has a lower diagnostic accuracy for distal DVT [20]. Symptomatic PE was diagnosed as the acute onset of dyspnea, chest pain, or syncope coupled with a new high-probability ventilation/perfusion lung scan, a new contrast filling defect on the spiral computed tomography or pulmonary angiography, or a new documentation of a proximal DVT either by ultrasound or venography. The ethics committees at each site approved the study (Northwest/Central Switzerland, Bern, Geneva, Eastern Switzerland, Vaud, Zurich; see http://www.swissethics.ch/eks.html for details). Eligible patients who had no exclusion criteria were approached for informed consent to participate in the study.

### Exclusion criteria

SWITCO65+ screened 1863 VTE patients (**S1 Fig**). Patients were excluded from the cohort due to inability to provide consent (n = 285), no consent (n = 398), follow-up not possible (n = 192), insufficient proficiency in German or French (n = 51), thrombosis at other sites than the lower limb (n = 21), and catheter-related thrombosis (n = 7). Multiple reasons for exclusion may apply. Patients with absent biosamples (n = 67), missing results from biosamples (n = 30), denying the use of data (n = 8), or withdrawing the informed consent within one day from inclusion (n = 4) were excluded from our analysis. Our study focuses on patients with a first VTE and we therefore excluded patients with prior VTE (n = 283).

### Baseline characteristics

The collection of biosamples and patient data was performed as previously described [15, 16]. In brief, trained research nurses collected blood samples at the time of index VTE. Blood processing was completed within 1 hour of collection. Data was recorded on standard data collection forms and included demographic information, comorbid conditions, medication history, and laboratory data. Clinical and laboratory characteristics at baseline are summarized in **Table 1**.

**Table 1:** Characterization of VTE patients.

Analyzed VTE patients	611 (100%)
**Demographic data**	
Age [years] (IQ-Range)	75 (69;81)
Females	278 (45%)
**VTE extent**	
Distal DVT only	51 (8%)
Proximal DVT ± distal DVT	133 (22%)
PE ± DVT	427 (70%)
**Medical history**	
Provoked index VTE	209 (34%)
Active cancer	127 (21%)
Severe infection/sepsis during the last 3 months	52 (9%)
Inflammatory bowel disease	18 (3%)
Acute rheumatic disease during the last 3 months	20 (3%)
Diabetes mellitus	97 (16%)
Arterial hypertension	388 (64%)
Chronic lung disease	80 (13%)
Chronic or acute heart failure	78 (13%)
History of major bleeding	63 (10%)
Anemia*	257 (42%)
Renal failure (GFR < 30ml/min)	35 (6%)
Body mass index (IQ-range)	26.2 (24.0;29.7)
**Medication**	
Concomitant antiplatelet therapy	194 (32%)
Initial vitamin K antagonist therapy	523 (86%)
Initial parenteral anticoagulation	590 (97%)
**Laboratory parameters**	
Leukocytes [K/μl] (IQ-Range)*	8.3 (6.5;11.1)
Platelets [K/μl] (IQ-Range)*	213 (168;275)
us-CRP [mg/L] (IQ-Range)*	24.4 (8.5;63.9)
D-dimer [ng/mL] (IQ-Range)*	2517 (1634;3784)

Baseline characteristics of analyzed patients at index VTE. Medication indicates the type of therapy applied upon diagnosis of acute VTE. Data presented as n (%) or median with interquartile (IQ)-range.

*Missing values: Anemia (n = 33), Renal Failure (n = 43), BMI (n = 5), Leukocytes (n = 34), Platelets (n = 32), us-CRP (n = 3), D-dimer (n = 21).

### Adverse outcomes of VTE

Adverse outcomes of VTE were defined as previously published [16]. The primary medical outcome was the recurrence of an objectively confirmed, symptomatic VTE, defined as a fatal or new non-fatal PE or new DVT (proximal and/or distal) based on previously published criteria [21, 22]. Additional adverse outcomes tested are the occurrence of all-cause, PE-related or -unrelated, and cancer-related or -unrelated mortality. Death was PE-related if PE was confirmed by autopsy, or if death followed a clinically severe PE, either initially or after an objectively confirmed recurrent event. Death in a patient who died suddenly or unexpectedly was classified as possibly PE-related. Unrelated deaths were classified as a result of an obvious cause other than PE [23]. A committee of three blinded clinical experts adjudicated all outcomes and classified the cause of all deaths as definitely due to PE, possibly due to PE or due to other causes. Final classifications were made on the basis of the full consensus of the committee.

### Quantification of ceDNA

CeDNA in plasma was quantified with modifications as previously described [24]. In brief, citrated plasma was diluted 20-fold in dilution buffer containing 0.1% BSA and 5 mM EDTA in phosphate-buffered saline (PBS). Fifty microliters of diluted plasma was mixed with 50 μl of PBS containing a fluorescent DNA-intercalating dye. DNA-fluorescence was then recorded in a multi plate fluorometer (Fluoroskan; Thermo Fisher Scientific). Autofluorescence was considered background and determined in samples mixed with PBS without DNA dye. In initial experiments, we compared PicoGreen (1%; Life Technologies) and SytoxGreen (1 μM; Life Technologies), two fluorescent DNA dyes commonly used for the quantification of extracellular DNA in plasma. While both dyes quantified DNA with a similar detection limit in saline, SytoxGreen displayed a significantly higher sensitivity for DNA in plasma (**S2 Fig**). We therefore used SytoxGreen as a probe for ceDNA quantification.

### Quantification of circulating nucleosomes

The quantification of circulating nucleosomes is based on a monoclonal antibody against an epitope on the complex of DNA with histone H2A and H2B [12]. We coated microtiter plates with 5 μg of this antibody. Plates were blocked with 2% bovine serum albumin (BSA, Sigma-Aldrich) and 1% dry milk powder (Spinnrad) for 1 hour at 37°C and washed once with PBS-Tween (PBS-T). Plasma samples, which were diluted 10-fold in PBS, were added to wells and incubated for 30 minutes at 37°C. Wells were then washed three times with PBS-T. For detection of DNA, we added PBS containing 1% of Picogreen. Fluorescence was then recorded with a multi plate fluorometer.

### Quantification of circulating NETs

We quantified complexes of DNA, histones, and myeloperoxidase (MPO) as a marker of NETs in plasma [25]. In brief, we coated microtiter plates with 1 μg of the anti-DNA-histone H2A/H2B-complex antibody [12]. Plates were blocked with 2% BSA and 1% milk powder for 1 hour at 37°C. Plasma was diluted 20-fold in dilution buffer containing 2 mM EDTA and 0.1% BSA in PBS. Diluted plasma was added to wells and incubated for 1 hour at 37°C. Wells were washed and then incubated for 15 minutes at 37°C with 1 μg/ml rabbit anti-human-MPO antibody (Dako) in PBS supplemented with 0.1% BSA. Following an additional washing step, anti-MPO-antibodies were detected by incubating wells with 0.1 μg/ml horseradish peroxidase (HRP)-conjugated goat antibodies against rabbit IgG (Santa Cruz) for 15 minutes at 37°C. After washing, HRP-activity was detected using ABTS solution (Life Technologies). NETs were isolated from activated neutrophils as previously described [26] and used as a positive control.

### Statistical analysis

We compared the concentration of ceDNA, circulating nucleosomes, and NETs in patient plasma by type of the index VTE using a nonparametric test for trend across ordered groups according to Cuzick [27]. For associations between the concentration of ceDNA and death, an ordinary Cox-regression with robust standard errors was calculated. For PE-related, non-PE-related, cancer-related, and non-cancer-related death, a competing risk regression was performed accounting for other causes of death as a competing event, according to the method of Fine and Gray [27]. We adjusted the models for risk factors that had been previously shown to be associated with the outcomes [28]. The method yields subhazard ratios (SHR) with corresponding 95% confidence intervals and p-values for the failure event of primary interest. VTE recurrence was adjusted for age, gender, cancer, provoked index VTE, and periods of oral or parenteral anticoagulation as a time-varying covariate. Mortality was adjusted for age, gender, overt PE, cancer, immobilization, heart failure, chronic lung disease, us-CRP, and periods of anticoagulation as a time-varying covariate. Cancer- and non-cancer-related mortality was adjusted for age, gender, overt PE, immobilization, chronic lung disease, us-CRP, and periods of anticoagulation as a time-varying covariate. Due to low number of events, PE- and non-PE-related mortality was only adjusted for age, overt PE, cancer, us-CRP, and periods of anticoagulation as a time-varying covariate. The discriminative ability of ceDNA for VTE recurrence and mortality was assessed by Harrell’s C concordance statistic. The prognostic ability for 3-month mortality was assessed by the area under the ROC curve (AUC). The added predictive ability of ceDNA combined with other biomarkers was assessed by the integrated discrimination improvement (IDI) index, which is based on a logistic model and was calculated by the method of Pencina et al [28]. All analyses were done using Stata 14 (Stata Corporation, College Station, Texas).

## Results

### Circulating extracellular DNA indicates the extent of VTE in patients

**Fig 1:**
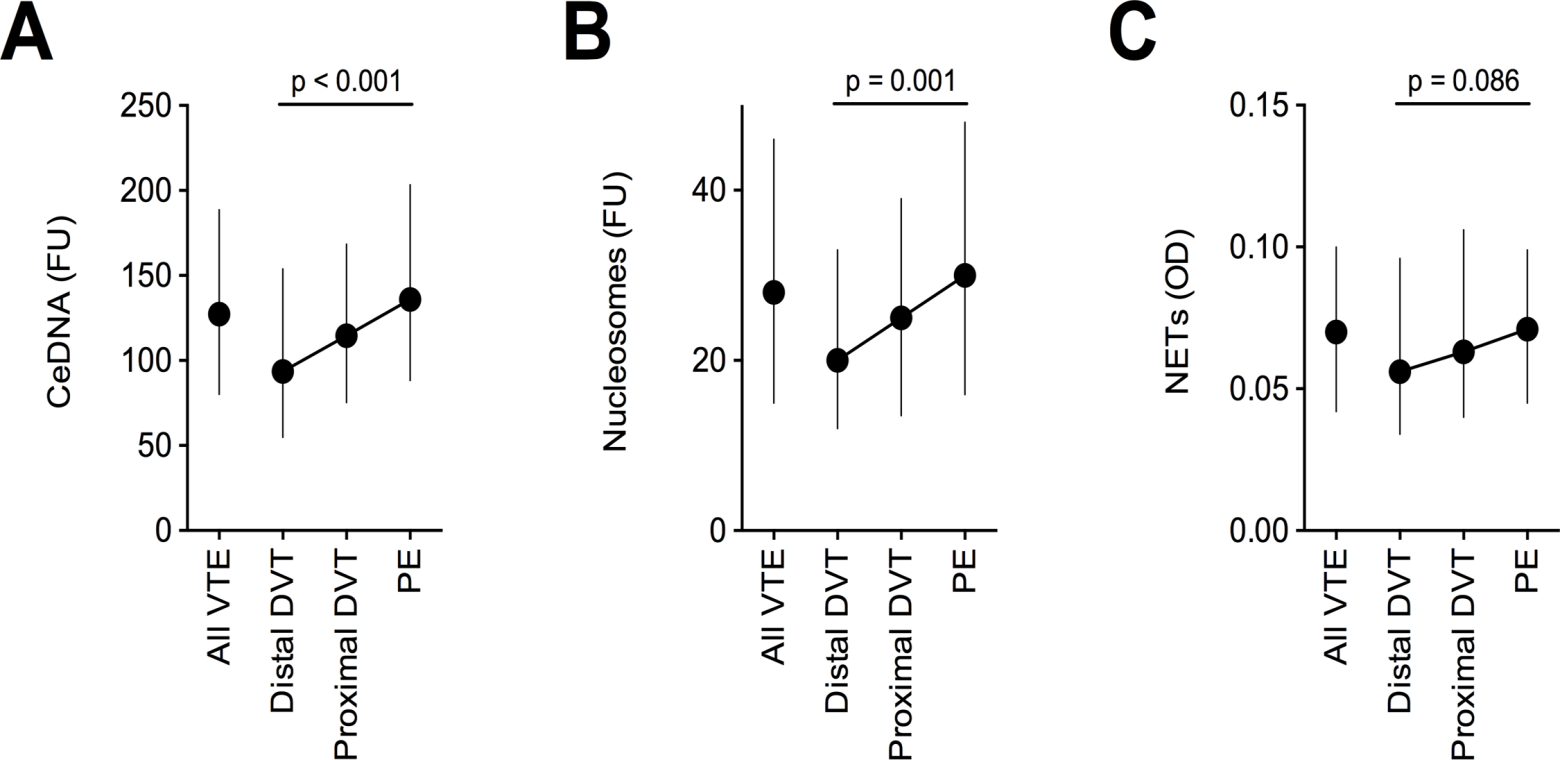
Association of ceDNA, circulating nucleosomes, and NETs with VTE extent. **(A)** CeDNA and (**B**) circulating nucleosomes in plasma correlated with the extent of VTE. (**C**) Plasma levels of the NET-specific biomarker, DNA-histone-MPO complexes, did not significantly correlate with VTE extent. Extent of acute VTE was categorized as isolated distal DVT only (n = 51), proximal DVT ± distal DVT (n = 133), and PE ± DVT (n = 427). All VTE (n = 611). FU: Fluorescent units; OD: Optical density. Data is shown as median ± inter-quartile range. P-values were calculated using a nonparametric test for trend across ordered groups.

We used a fluorescent probe to quantify ceDNA in citrated plasma from patients of the SWITCO65+ study. The cohort enrolled patients aged 65 years or older with acute, symptomatic VTE (**Table 1**). Our study focuses on patients with a first acute VTE event and therefore excluded patients with prior VTE (**S1 Fig**). We analyzed 611 VTE patients including 51 patients with distal DVT only, 133 patients with proximal DVT, and 427 patients with PE (**Table 1**). The levels of ceDNA increased with the extent of VTE categorized as isolated distal DVT, proximal DVT, and PE (**Fig 1A**). To investigate the potential origin of ceDNA, we correlated the levels of ceDNA, with levels of circulating nucleosomes, and NETs. We used an antibody against an epitope on the complex of DNA, histone H2A, and histone H2B to immunoabsorb and quantify nucleosomes. NETs, which are characterized as chromatin fibers containing enzymes from neutrophil granules [12], were quantified by detection of the complex of DNA, histones, and myeloperoxidase. We observed a strong correlation of ceDNA and circulating nucleosomes (Spearman r = 0.656, p < 0.001), indicating that ceDNA in VTE patients is circulating in part as nucleosomes. Levels of ceDNA and NETs did not correlate (Spearman r = 0.057, p = 0.159), suggesting that NETs are not a major component of ceDNA in VTE patients. In line with these results, levels of ceDNA (z-statistics: 4.26; p < 0.001) and circulating nucleosomes (3.48; p = 0.001), but not NETs (1.72; p = 0.086) correlated significantly with the extent of VTE (**Fig 1B and Fig 1C**).

We previously observed that high concentrations of anticoagulants, in particular heparins, disintegrate NETs *in vitro* [29]. We analyzed the effect of anticoagulation (AC; parenteral or Vitamin K antagonist) on ceDNA levels in acute VTE patients. AC was given to 362 patients more than one day before blood sampling, to 139 patients one day before blood sampling, to 105 patients after blood samples, while 5 patients did not receive AC. We observed no significant association between ceDNA and timing of AC start (Kruskal-Wallis test; p = 0.24), suggesting that AC therapy has no major effect of ceDNA levels. These data furthermore support that NETs are not a major source of ceDNA in acute VTE patients.

### Circulating extracellular DNA predicts mortality after acute VTE

**Fig 2:**
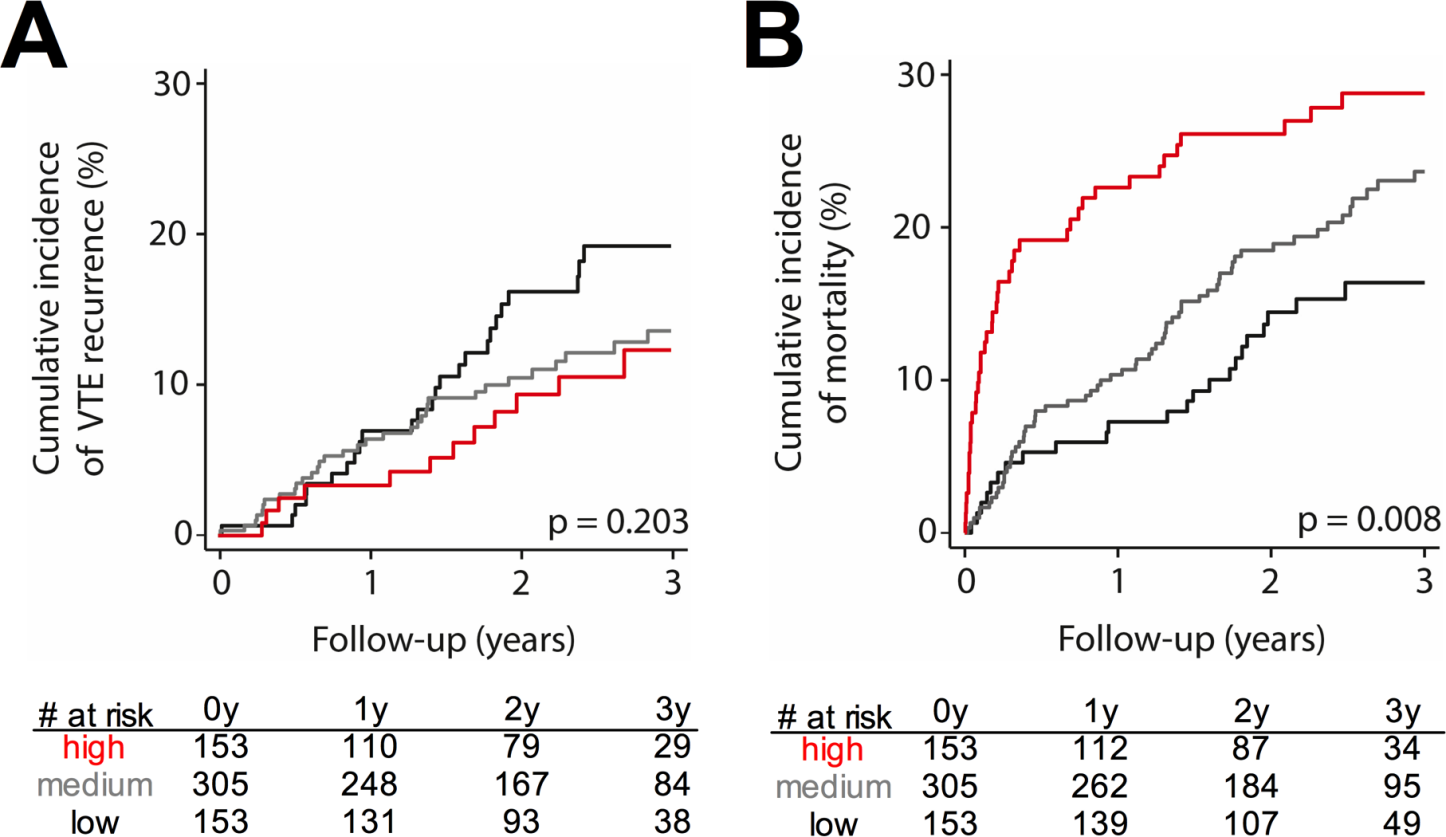
Association of ceDNA with the outcome following acute VTE. Kaplan-Meier-curves show the cumulative incidence of (**A**) VTE recurrence and (**B**) mortality. Patients were grouped according to the concentration of ceDNA at the time of acute VTE into high levels (4^th^ quartile, red line), medium levels (2^nd^-3^rd^ quartile, grey line), and low levels (1^st^ quartile, black line). VTE patients with high ceDNA have an increased risk of mortality, but not VTE recurrence. P-values were calculated using a logrank test.

We analyzed a possible association of ceDNA with the clinical outcome following acute VTE. The median follow-up time was 29.7 months (IQ-range: 18.2–40.7). We trichotomized VTE patients based on their concentration of ceDNA at the time of acute VTE, choosing the 75^th^ percentile, and 25^th^ percentile as pre-specified cut-off values. Seventy patients suffered recurrent VTE during follow-up. We did not observe an association of increased ceDNA levels with VTE recurrence (**Fig 2A**). Analyzing continuous rather than trichotomized ceDNA levels showed similar results (**Table 2**). 129 patients died during follow-up. Analysis of trichotomized (**Fig 2B**) and continuous data (**Table 2**) revealed that elevated levels of ceDNA at acute VTE strongly increase the risk of mortality. Forty-one of 611 patients died within 3 months after acute VTE and calculation of hazard ratios (HR) and adjusting for several confounders revealed that ceDNA was significantly associated with an approximately 2.5-fold increase in risk of death (HR: 2.57, 95%-CI 1.59–4.15; **Table 3**). The cause of death was related to PE in 9 patients (**S1 Table**). Elevated levels of ceDNA were associated with an approximately 3.5-fold increased risk of fatal PE (HR: 3.68; 95%-CI 1.70–7.94) and 2-fold increased risk of non-PE-related causes of death (HR: 1.85, 95%-CI 1.27–2.71; **Table 3**).

**Table 2:** Association of ceDNA with VTE recurrence and mortality during follow-up.

	n	C-statistics	95% CI	p-value
VTE recurrence				
Up to 3 months	4	0.34	0.18–0.50	0.056
Up to 12 months	32	0.45	0.35–0.54	0.284
Up to 24 months	60	0.44	0.37–0.52	0.133
Up to 36 months	70	0.45	0.38–0.52	0.144
Mortality				
Up to 3 months	41	0.71	0.61–0.80	< 0.001
Up to 12 months	76	0.65	0.58–0.72	< 0.001
Up to 24 months	114	0.61	0.55–0.66	< 0.001
Up to 36 months	129	0.60	0.55–0.65	< 0.001

CeDNA was not significantly associated with VTE recurrence, but strongly associated with all-cause mortality. CeDNA was used as continuous variable; n: number of events. 95% CI, 95% confidence interval. P-values were calculated for the C-statistics. A C-statistics value of 0.5 indicates no predictive value (null hypothesis).

**Table 3:** Association of log-transformed ceDNA with 3-month mortality.

		crude			adjusted		
	n	HR or SHR	95% CI	p-value	HR or SHR	95% CI	p-value
All-cause mortality	41	2.65	1.79–3.92	< 0.001	2.57	1.59–4.15	< 0.001
PE-related death	9	3.62	2.10–6.23	< 0.001	3.68	1.70–7.94	0.001
Non-PE-related death	32	2.03	1.43–2.90	< 0.001	1.85	1.27–2.71	0.002
Cancer-related death	15	2.36	1.54–3.60	< 0.001	2.43	1.46–4.03	0.001
Non-cancer-related death	26	2.50	1.56–4.01	< 0.001	2.26	1.27–4.01	0.005

Crude and adjusted subhazard ratios (SHR) with 95% confidence intervals, and corresponding p-values. In case of overall mortality, the hazard ratio (HR) is shown (no competing risk). HR/SHRs are expressed per one log-unit increase in ceDNA. Adjustment was done for age, gender, overt PE, cancer, immobilization, heart failure, chronic lung disease, periods of anticoagulation, and us-CRP as a time-varying covariate. Due to low number of events, PE- and non-PE-related mortality was only adjusted for age, overt PE, and periods of anticoagulation as a time-varying covariate. Cancer- and non-cancer-related mortality was adjusted for age, gender, overt PE, immobilization, chronic lung disease, periods of anticoagulation, and us-CRP as a time-varying covariate.

Levels of ceDNA are elevated in various conditions [5]. We analyzed whether comorbid conditions in our VTE patients are associated with increased levels of ceDNA. Adjusting for age, sex, and all other comorbidities in a multivariate linear regression revealed that an accompanying or preceding severe infection or sepsis, but not cancer, was independently associated with increased levels of ceDNA in VTE (**Table 4**). In line with these results, the association of ceDNA with cancer-related mortality (HR: 2.43; 95%-CI 1.46–4.03) and non-cancer-related mortality (HR: 2.26; 95%-CI 1.27–4.01) was comparable (**Table 3)**.

**Table 4:** Association of ceDNA with comorbidities.

		crude			adjusted		
	n	CR	95% CI	p-value	AR	95% CI	p-value
Severe infection/sepsis	52	1.43	1.19–1.72	< 0.001	1.34	1.11–1.63	0.003
Active cancer	127	1.18	1.04–1.34	0.011	1.09	0.95–1.24	0.234
Arterial hypertension	388	1.06	0.95–1.18	0.274	1.10	0.98–1.23	0.114
Diabetes mellitus	97	1.02	0.89–1.18	0.744	0.98	0.84–1.14	0.785
Chronic lung disease	80	0.97	0.83–1.13	0.678	0.90	0.77–1.06	0.211
Chronic or acute heart failure	78	1.03	0.88–1.20	0.722	1.00	0.85–1.18	0.981
Inflammatory bowel disease	18	1.04	0.77–1.41	0.803	0.96	0.70–1.31	0.788
Acute rheumatic disease	20	0.99	0.74–1.32	0.935	1.02	0.76–1.37	0.886
Major surgery	106	1.16	1.01–1.33	0.036	0.99	0.85–1.17	0.945
Renal failure	35	1.11	0.89–1.39	0.351	1.10	0.87–1.40	0.408

Crude and adjusted robust linear regression of log-transformed ceDNA on comorbidities. Adjustment was done for age, gender, severe infection/sepsis, active cancer, arterial hypertension, diabetes mellitus, chronic lung disease, chronic or acute heart failure, inflammatory bowel disease, acute rheumatic disease, major surgery, renal failure (GFR < 30 ml/min), estrogen therapy, immobilization, history of major bleeding, anemia, concomitant antiplatelet therapy, and anticoagulation prior to index VTE. Coefficients were back-transformed by exponentiation yielding ratios. CR: crude ratio; AR: adjusted ratio.

### Circulating extracellular DNA is associated with markers of acute inflammation

**Fig 3:**
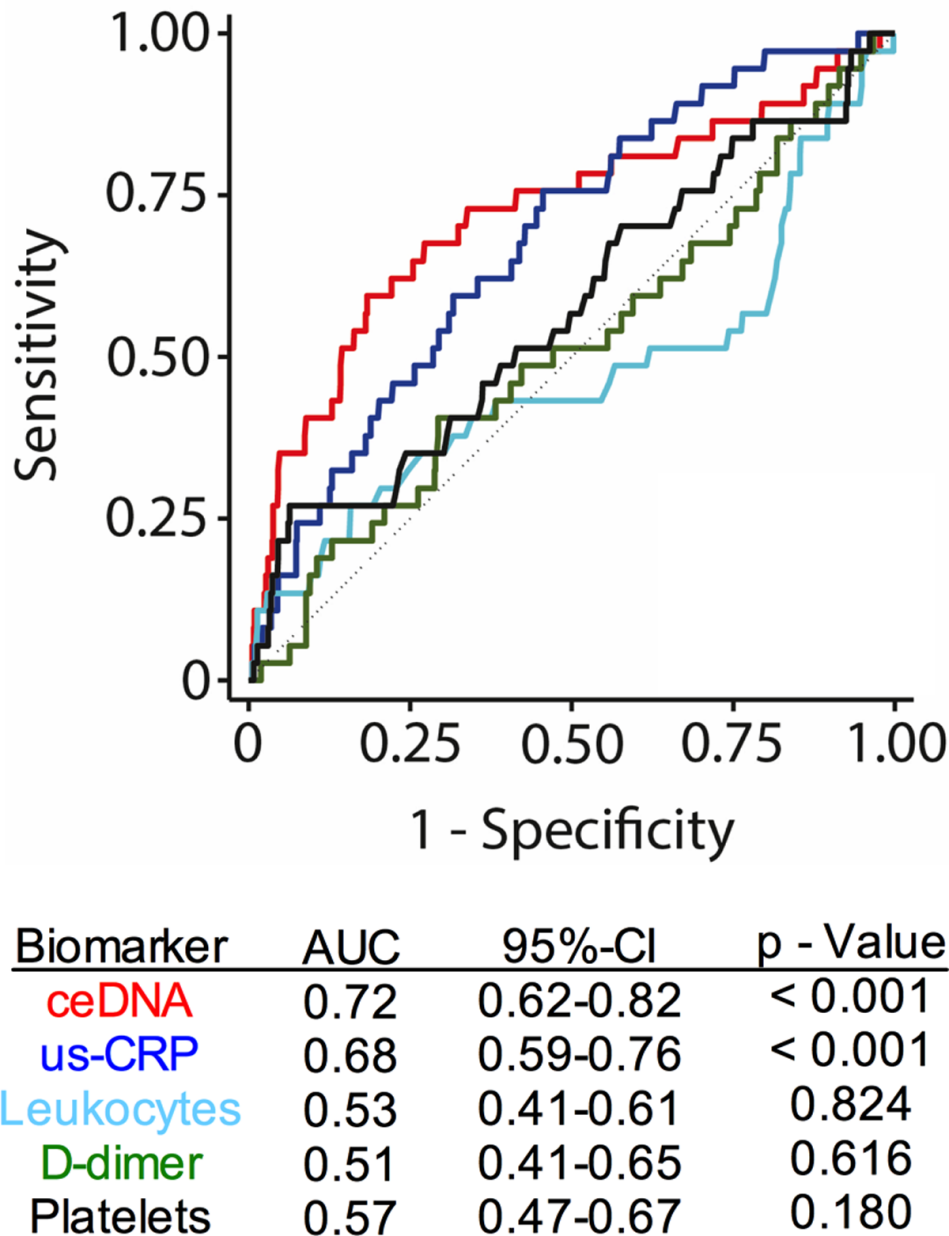
Comparison of the ceDNA to laboratory markers of inflammation and thrombosis for the prediction of 3-month-mortality. Area under the curve (AUC) from ROC analysis of ceDNA, us-CRP, leukocyte count, D-dimer, platelet count shows ceDNA and usCRP to be strong predictors of 3-months mortality following acute VTE.

We questioned whether ceDNA is associated with other established markers of thrombosis and inflammation. We used platelet counts and levels of the fibrin degradation product D-dimer as surrogate markers for thrombosis. Levels of ultra-sensitive C-reactive protein (us-CRP) and white blood cell counts were used as biomarkers of acute inflammation. We observed no or a weak correlation of ceDNA with platelets (Spearman r: 0.05; p = 0.252) and D-dimer (r: 0.08; p = 0.041), respectively. CeDNA did strongly correlate with us-CRP (r: 0.37; p < 0.001) and leukocytes (r: 0.23; p < 0.001), suggesting that increased levels of ceDNA are associated with acute inflammation in VTE patients. A statistical comparison of biomarkers revealed that ceDNA and us-CRP, but not leukocytes, platelets or d-dimer, significantly predicted death following acute VTE (**Fig 3**). Furthermore, ceDNA was associated with an increased risk of mortality independently of other biomarkers tested, and the combination of ceDNA and us-CRP predicted mortality more accurately than each marker alone (**S2 Table**). In conclusion, our study shows that ceDNA is a strong and independent predictor of mortality following acute VTE.

## Discussion

In 1973, Davis and colleagues first identified ceDNA in plasma from patients with PE [4]. A number of subsequent studies confirmed the increased levels of ceDNA in DVT and PE patients compared to healthy controls [5]. CeDNA exists in different forms including mitochondrial DNA (mtDNA), nuclear DNA (nDNA), nucleosomes, consisting of nDNA in complex with histones, as well as NETs [5]. Current methods detecting ceDNA employ DNA-specific fluorescent dyes, qPCR, and ELISA. Quantification of nDNA- and mtDNA-specific DNA sequences in plasma from 74 PE patients revealed that patients with massive PE have higher ceDNA levels than patients with sub-massive PE [30]. Higher levels of nDNA and mtDNA furthermore correlate with increased risk for mortality within 15 days [30]. Similarly, levels of circulating nucleosomes and ceDNA are increased in patients with DVT when compared to patients with clinical suspicion of DVT, but in whom DVT was excluded [13, 14].

We analyzed the association of ceDNA in plasma with the extent and outcome of acute VTE. Our study focused on the detection of ceDNA by fluorescent dyes. This method is automatable, cost-effective, and provides rapid results and thus may be used in a clinical diagnostic setting in future. We observed that levels of ceDNA strongly correlated with VTE extent and predicted mortality, but not VTE recurrence. Furthermore, we identified a strong association of ceDNA with markers of laboratory markers of acute inflammation. Our study focuses on acute VTE in elderly patients, the epidemiologically most relevant age group [3]. We have analyzed the levels of ceDNA in more than 600 patients. To our knowledge, our study is the largest analysis of ceDNA in patients to date. The evaluation of a large prospective cohort identified that non-infectious comorbidities including cancer do not independently increase ceDNA levels in acute VTE. This suggests that the increase of ceDNA levels is mainly a consequence of acute VTE. The source of ceDNA in VTE patients is unknown. NETs are abundantly present in thrombi from VTE patients [31]. Furthermore, in animal models, the formation of NETs during thrombosis timely coincides with an elevation in ceDNA [29]. However, NETs levels in plasma neither correlated with ceDNA nor with VTE extent, suggesting that NETs are not the main source of ceDNA in VTE patients. Therefore, it is conceivable that ceDNA derives from injured tissue, which is damaged due to the thrombotic occlusion of supplying blood vessels. Indeed, ceDNA levels correlate with markers of cell injury and size of ischemic lesions in several cardiovascular and thromboembolic diseases, including myocardial infarction, stroke, and venous thromboembolism [5]. DNA, histones, or other damage-associated molecular patterns (DAMPs) may initiate an inflammatory response via activating pattern-recognition receptors on immune cells [32]. In line with this hypothesis, we observed a strong correlation of ceDNA with markers of acute inflammation, namely us-CRP and circulating leukocytes. Increased levels of ceDNA may indicate the degree of systemic inflammation, which is associated with shortened survival after a VTE event. In conclusion, ceDNA may help identifying VTE patients with a poor prognosis and improve the clinical management of VTE in the future.

## Supporting information

S1 Fig Patient recruitment chart with reasons for exclusions. (DOCX)

S2 Fig Comparison of ceDNA quantification using the DNA-intercalating dyes PicoGreen or SytoxGreen. (A) Detection of DNA in PBS. PicoGreen and SytoxGreen detect DNA in PBS with a similar sensitivity (2-way ANOVA with Bonferroni posttest; * p < 0.05, **** p < 0.0001 compared to 0 ng/ml DNA in PBS). (B) Detection of DNA in plasma. Plasma from healthy control donors was supplemented with indicated concentrations of DNA. SytoxGreen displays a higher sensitivity to detect plasma DNA than PicoGreen (2-way ANOVA with Bonferroni posttest; ** p < 0.01, **** p < 0.0001 compared to 0 ng/ml DNA in plasma). Data was normalized to the fluorescence intensity at 0 ng/ml DNA in (A) PBS or (B) plasma. Data shown as mean ± SD, n = 3.
(DOCX)

S1 Table List of causes of death at 3-months follow-up. (DOCX)

S2 Table Prognostic accuracy of ceDNA for 3-months mortality. (DOCX)
