# Supplementary figures and images for: Circulating extracellular DNA is an independent predictor of mortality in elderly patients with venous thromboembolism
Source: PLoS One. 2018 Feb 23;13(2):e0191150. doi: 10.1371/journal.pone.0191150 (PMC5825008; doi:10.1371/journal.pone.0191150)

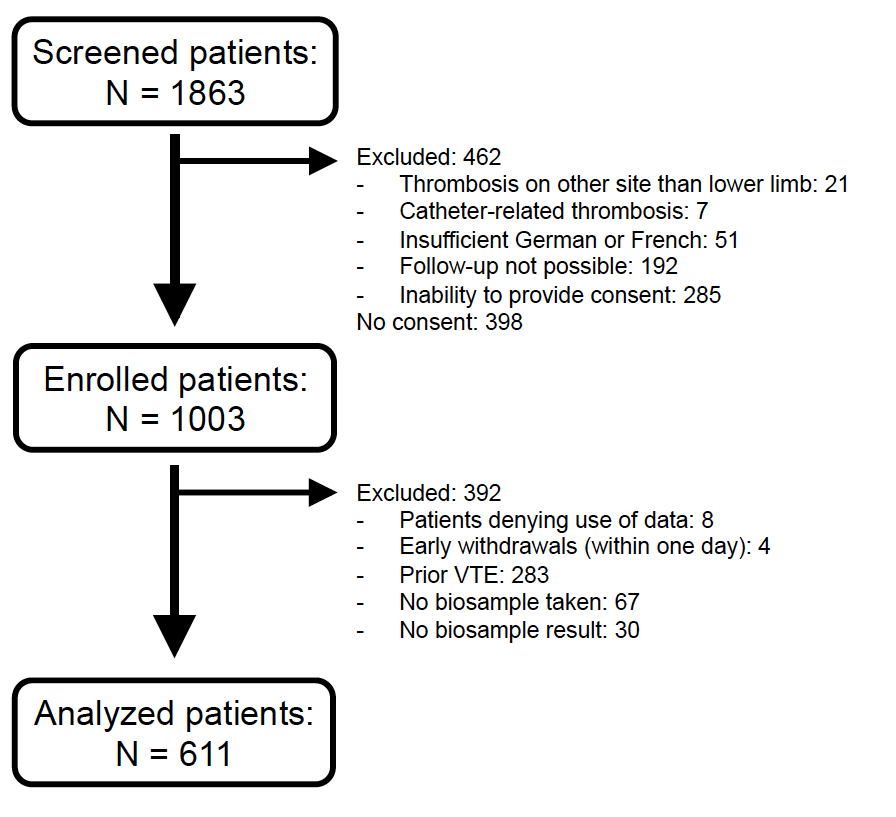


**S1 Fig**. **Patient recruitment chart**. Patient recruitment chart with reasons for exclusions

Supplement: S1 Fig — (DOCX) [file pone.0191150.s001.docx]
