# Supplementary material for: Circulating extracellular DNA is an independent predictor of mortality in elderly patients with venous thromboembolism
Source: PLoS One. 2018 Feb 23;13(2):e0191150. doi: 10.1371/journal.pone.0191150 (PMC5825008; doi:10.1371/journal.pone.0191150)
Supplement: S2 Fig — (A) Detection of DNA in PBS. PicoGreen and SytoxGreen detect DNA in PBS with a similar sensitivity (2-way ANOVA with Bonferroni posttest; * p < 0.05, **** p < 0.0001 compared to 0 ng/ml DNA in PBS). (B) Detection of DNA in plasma. Plasma from healthy control donors was supplemented with indicated concentrations of DNA. SytoxGreen displays a higher sensitivity to detect plasma DNA than PicoGreen (2-way ANOVA with Bonferroni posttest; ** p < 0.01, **** p < 0.0001 compared to 0 ng/ml DNA in plasma). Data was normalized to the fluorescence intensity at 0 ng/ml DNA in (A) PBS or (B) plasma. Data shown as mean ± SD, n = 3. (DOCX) [file pone.0191150.s002.docx]

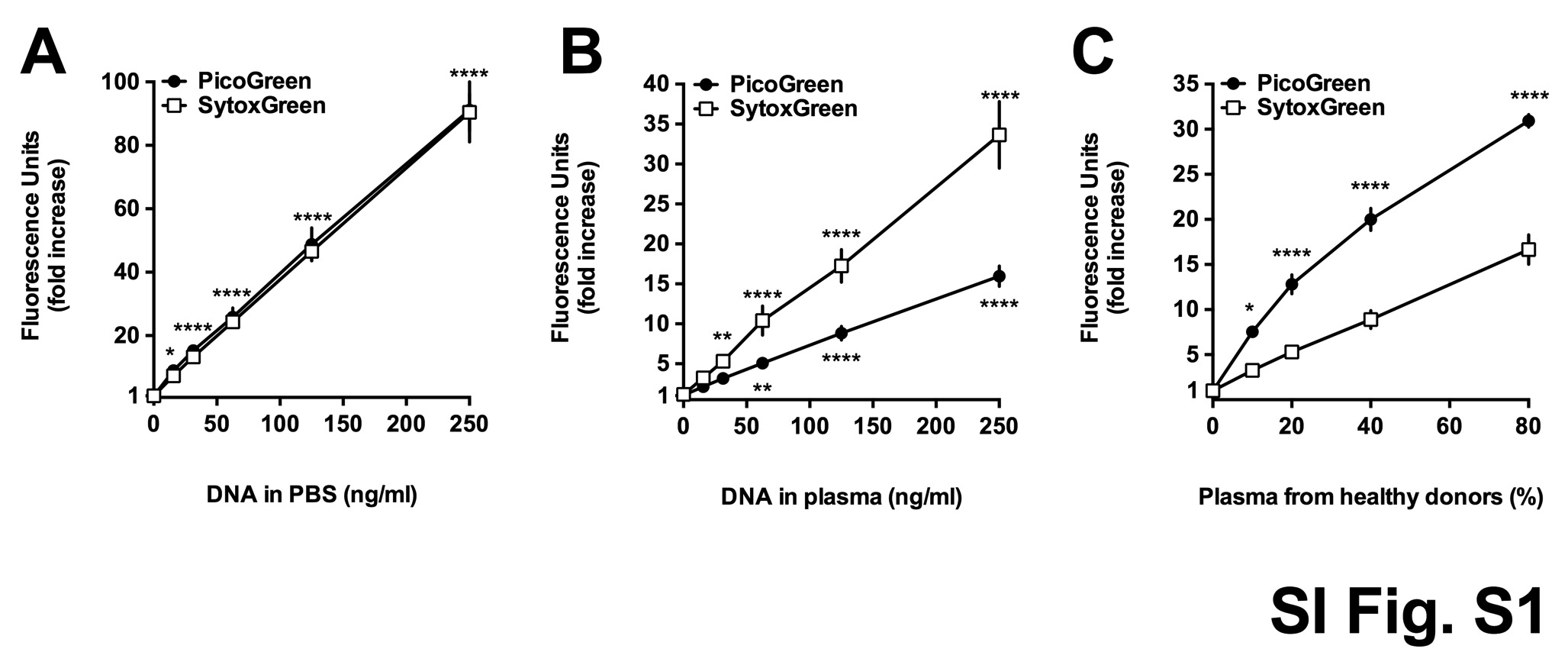


**S2 Fig**. **Comparison of ceDNA quantification using the DNA-intercalating dyes PicoGreen or SytoxGreen.** (A) Detection of DNA in PBS. PicoGreen and SytoxGreen detect DNA in PBS with a similar sensitivity (2-way ANOVA with Bonferroni posttest; * p < 0.05, **** p < 0.0001 compared to 0 ng/ml DNA in PBS). (B) Detection of DNA in plasma. Plasma from healthy control donors was supplemented with indicated concentrations of DNA. SytoxGreen displays a higher sensitivity to detect plasma DNA than PicoGreen (2-way ANOVA with Bonferroni posttest; ** p < 0.01, **** p < 0.0001 compared to 0 ng/ml DNA in plasma). Data was normalized to the fluorescence intensity at 0 ng/ml DNA in (A) PBS or (B) plasma. Data shown as mean ± SD, n = 3.
