# Supplementary material for: Circulating extracellular DNA is an independent predictor of mortality in elderly patients with venous thromboembolism
Source: PLoS One. 2018 Feb 23;13(2):e0191150. doi: 10.1371/journal.pone.0191150 (PMC5825008; doi:10.1371/journal.pone.0191150)
Supplement: S1 Table — (DOCX) [file pone.0191150.s003.docx]

**S1 Table. List of causes of death at 3-months follow-up**.

| Cause of death |  | n | | |
| --- | --- | --- | --- | --- |
| All |  | 41 (100%) | | |
| Cancer |  | 15 (36.6%) | | |
| Possibly PE-related |  | 6 (14.6%) | | |
| PE-related |  | 3 (7.3%) | | |
| Bleeding |  | 2 (4.9%) | | |
| Infection |  | 3 (7.3%) | | |
| Sepsis |  | 3 (7.3%) | | |
| Pulmonary causes other than PE |  | 2 (4.9%) | | |
| Left ventricular failure |  | 2 (4.9%) | | |
| Acute coronary syndrome |  | 1 (2.4%) | | |
| Unknown |  | 4 (9.8%) | | |
|  |  | |  |  |

Of 41 deaths during follow-up, 9 deaths were related or possibly related to PE.
