# Supplementary material for: Circulating extracellular DNA is an independent predictor of mortality in elderly patients with venous thromboembolism
Source: PLoS One. 2018 Feb 23;13(2):e0191150. doi: 10.1371/journal.pone.0191150 (PMC5825008; doi:10.1371/journal.pone.0191150)
Supplement: S2 Table — (DOCX) [file pone.0191150.s004.docx]

**S2 Table. Prognostic accuracy of ceDNA for 3-months mortality.**

|  | AUC | 95% CI | p-value |  | IDI | 95% CI | p-value |
| --- | --- | --- | --- | --- | --- | --- | --- |
| ceDNA | 0.72 | 0.62-0.82 | < 0.001 |  | - | - | - |
| us-CRP | 0.68 | 0.59-0.76 | < 0.001 |  | 0.046 | 0.019-0.073 | 0.001 |
| Leukocytes | 0.53 | 0.41-0.65 | 0.616 |  | 0.062 | 0.028-0.096 | <0.001 |
| D-dimer | 0.51 | 0.41-0.61 | 0.824 |  | 0.058 | 0.025-0.090 | <0.001 |
| Platelets | 0.57 | 0.47-0.67 | 0.180 |  | 0.058 | 0.026-0.091 | <0.001 |
|  |  |  |  |  |  |  |  |

Complete analysis was performed using only patients with complete data on all biomarkers (555 patients, 37 events). The prognostic value was assessed by the area under the ROC curve (AUC). The added predictive ability of ceDNA combined with other biomarkers was assessed by the integrated discrimination improvement (IDI) index.
